# Recurrent carotid artery stenosis successfully and safely treated with drug-coated balloon angioplasty under flow reversal

**DOI:** 10.1016/j.jvscit.2025.101728

**Published:** 2025-01-15

**Authors:** Marianna Pavlyha, Steven Farley, Wesley S. Moore

**Affiliations:** Division of Vascular and Endovascular Surgery, Department of Surgery, University of California Los Angeles, Los Angeles, CA

**Keywords:** Carotid restenosis, TCAR, Drug-coated balloon angioplasty, Flow reversal

## Abstract

Carotid artery restenosis after index carotid artery revascularization reduces its stroke prevention benefit. A 73-year-old woman presented with recurrent right carotid artery restenosis following two carotid endarterectomies with patch angioplasty and in-sent restenosis after subsequent transcarotid artery revascularization. We performed in-stent paclitaxel-coated balloon angioplasty under flow reversal with resolution of the lesion on imaging and improvement in symptoms. Patient remains asymptomatic with no evidence of restenosis 16 months after treatment.

Carotid revascularization is widely used for the treatment of both symptomatic and severe asymptomatic carotid artery stenosis for prevention of ischemic stroke. It has been reported that anywhere from 6% to 36% of patients will develop restenosis after carotid endarterectomy (CEA) with or without patching,^1^ and 3.8% to 23% in-stent restenosis after transfemoral stenting.^2^ Data on restenosis after transcarotid artery revascularization (TCAR) is not as robust, but a small study reports 18% restenosis rate within the median follow-up of 345 days.^3^ Both redo endarterectomy and angioplasty/stenting are option when intervention is indicated.^4^^,^^5^ Drug-coated balloon (DCB) angioplasty has recently been used more frequently for the treatment of recurrent stenosis after CEA and in-stent restenosis^6^^,^^7^^;^ however, there are no well-established guidelines that outline endovascular treatment of these lesions in the literature. We present a patient with recurrent right carotid artery restenosis after two open carotid interventions and in-sent restenosis after subsequent TCAR, who was successfully treated with DCB angioplasty using flow reversal for cerebral protection. The patient provided consent to publish their case details and images.

## Case Report

A 73-year-old woman with a history smoking, hypertension, dyslipidemia, and right hemispheric stroke with left-sided hemiparesis underwent a right CEA with patch angioplasty 3 months after the initial neurological event. During the course of her follow-up, she developed recurrent stenosis with symptoms, and therefore underwent a second CEA within patch angioplasty 1 year after the index operation. At that time she has quit smoking and her hypertension was well-controlled. She was compliant with high-dose statin and aspirin therapy. She had been doing well after the procedure; however, she was lost to follow-up and re-presented to the clinic 5 years later with global ischemic symptoms, which included dizziness, particularly with head turning. Otherwise she had no focal neurological events. Given that these symptoms were nonfocal, she underwent further work-up, including assessment for noncirculatory diagnoses. Imaging studies suggested a recurrent ipsilateral lesion at the junction of the bulb and internal carotid artery (ICA) with an estimated 60% to 79% stenosis and ICA/CCA ratio of 2.2. On subsequent imaging her ICA/CCA ratio progressed to 2.5, 2.9, and 3.5 (Table) with antegrade flow in bilateral vertebral arteries. At that time, she also started describing right-sided ocular symptoms with recurrent episodes of weakness in the left leg and severe dizziness. She has been normotensive to hypertensive throughout all her visits. Computed tomography angiography was performed, which showed progressive lesion in the right carotid artery (Figure, *A*). Intervention options (second reoperation, TCAR, or no intervention) were discussed with the patient, and she elected to proceed with TCAR. She underwent successful placement of a 10 mm × 30 mm ENROUTE stent (Silk Road Medical Inc, Sunnyvale, CA) in the right common and internal carotid arteries without any complications and resolution of symptoms and stenosis on postoperative imaging.

**Table:** Timeline of interventions and ultrasound duplex findings

Date	ICA PSV (cm/s)	ICA/CCA ratio
2004	Right CEA with patch angioplasty	
2005	Right CEA with patch angioplasty	
8/2016	167	1.9
2/2019	195	1.9
2/2020	105	2.3
8/2020	173	2.5
4/2021	82	2.9
10/2021	**259**	**3.5**
11/2021	Right TCAR	
12/2021	61	0.9
7/2022	223	2.6
11/2022	**232**	**4.0**
3/2023	**404**	**8.5**
3/2023	Right ICA in-stent DCB	
4/2023	125	1.0
10/2023	142	1.6
7/2024	145	2.1

*CCA,* Common carotid artery; *CEA,* carotid endarterectomy; *ICA,* internal carotid artery; *DCB,* drug-coated balloon; *PSV,* peak systolic velocity; *TCAR,* transcarotid artery revascularization.

**Fig:**
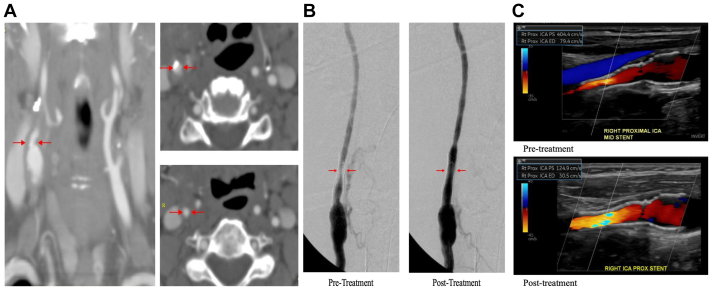
**(A)** Computed tomography angiogram of the neck before transcarotid artery revascularization (TCAR). Red arrows indicate area of right internal carotid artery (ICA) restenosis. **(B)** Intraoperative angiogram of right carotid artery before and after drug-coated balloon (DCB) angioplasty. Red arrows indicate area of restenosis. **(C)** Preoperative and postoperative duplex ultrasound examination of the right ICA. *PS*, peak systolic velocity; *Rt*, right.

Six months following the TCAR procedure, the patient again complained of episodes of dizziness. She underwent ultrasound duplex imaging, which now showed evidence of in-stent restenosis with an ICA/CCA ratio of 2.6, consistent with 60% to 79% stenosis. Interestingly, the patient was also noted to have a left subclavian artery stenosis with bidirectional flow in the left vertebral artery, which was a new finding. This was treated via a left transbrachial approach with a 9 × 19 mm Omnilink balloon expandable stent (Abbot Vascular, Abbott Park, IL) to restore antegrade flow to the left vertebral artery with excellent technical results. This resulted in partial symptom improvement for the patient; however, she continued to progress with her in-stent carotid lesion, with the ICA/CCA ratio increasing to 4.0 and later to 8.5. A decision was made to treat this with a 5 × 40 mm IN.PACT Admiral paclitaxel-DCB (Medtronic, Minneapolis, MN) under flow reversal using the ENROUTE Transcarotid Neuroprotection System (Silk Road Medical Inc) with stenosis resolution on completion angiogram (Figure, *B* and *C*). The patient's postoperative course was uneventful, and she was seen in clinic with complete resolution of her symptoms and normal follow-up duplex ultrasound showing ICA/CCA ratio of 1.0. It has been over a year since her last treatment and the patient continues to do well without any symptoms.

## Discussion

Restenosis after carotid artery intervention is a known complication, which leads to an increased risk of ipsilateral embolic events. Restenosis results as a consequence of neointimal hyperplasia, introduced both by endovascular and open techniques. Some risk factors that predispose patients to restenosis are prior operation or radiation to the ipsilateral neck, female sex, hyperlipidemia, and diabetes.^2^^,^^8^ Our patient exhibited at least two of these factors. The Society for Vascular Surgery^4^ and European Society for Vascular Surgery^5^ provide recommendations for management of carotid artery restenosis. When indicated, a redo endarterectomy or endovascular intervention is chosen based on multidisciplinary team review. However, there are no well-established guidelines that outline recommendations for endovascular treatment and choice of embolic protection, particularly in patients with multiple prior interventions and restenoses. Angioplasty with regular balloon and restenting have been the most frequently described modalites,^6^ with varied degrees of reported restenosis and a small risk of embolization.^9^^,^^10^ DCBs have shown good results for neointimal hyperplasia in peripheral cases,^11^ yet have not been adapted widely for the management of carotid disease. Thus far, studies on the use of DCB for treatment of carotid in-stent restenosis have reported good technical success and low complication rate.^7^^,^^12^^,^^13^ However, the main reservation with the use of DCB in the carotid distribution is the possible risk of the drug carrier leading to symptomatic cerebral embolization. A recent case series looking at the use of DCB angioplasty with or without stent placement and the Emboshield NAV (Abbot Vascular) embolic protection device showed promising results in asymptomatic patients.^14^ Similar good results with filter protection for asymptomatic severe in-stent restenosis were reported in another small case series.^15^ To our knowledge, we report the first successful treatment of recurrent in-stent restenosis with a combination of DCB and blood flow reversal through an ex vivo common carotid artery to femoral vein shunt using the ENROUTE Transcarotid Neuroprotection System (Silk Road Medical Inc)^16^^,^^17^ for distal embolic protection from balloon coating. Large studies with both short- and long-term follow-up periods are needed to further establish outcomes on the use of DCB for the treatment of recurrent carotid artery restenosis using flow reversal for protection against drug carrier embolization.

## Conclusions

We performed DCB angioplasty under flow reversal for carotid artery in-stent restenosis in a patient with two prior open interventions and subsequent TCAR with refractory restenosis. This resulted in a safe and successful treatment of the lesion and resolution of symptoms 16 months after treatment.

## Funding

None.

## Disclosures

None.
